# Fluorescence *in situ* hybridization and sequential catalyzed reporter deposition (2C-FISH) for the flow cytometric sorting of freshwater ultramicrobacteria

**DOI:** 10.3389/fmicb.2015.00247

**Published:** 2015-03-31

**Authors:** Stefan M. Neuenschwander, Michaela M. Salcher, Jakob Pernthaler

**Affiliations:** Limnological Station, Institute of Plant Biology, University of ZurichKilchberg, Switzerland

**Keywords:** flow cytometry, flow sorting, fluorescence *in situ* hybridization, catalyzed reporter deposition, immunohistochemistry, freshwater bacterioplankton, ultramicrobacteria

## Abstract

Flow cytometric sorting is a powerful tool to physically separate cells within mixed microbial communities. If combined with phylogenetic staining (fluorescence *in situ* hybridization, FISH) it allows to specifically sort defined genotypic microbial populations from complex natural samples. However, the targeted enrichment of freshwater ultramicrobacteria, such as members of the LD12 clade of *Alphaproteobacteria* (SAR11-IIIb), is still challenging. Current FISH protocols, even in combination with signal amplification by catalyzed reporter deposition (CARD), are not sufficiently sensitive for the distinction of these bacteria from background noise by flow cytometry, presumably due to their low ribosome content and small cell sizes. We, therefore, modified a CARD based flow sorting protocol with the aim of increasing its sensitivity to a level sufficient for ultramicrobacteria. This was achieved by a second signal amplification step mediated by horseradish peroxidase labeled antibodies targeted to the fluorophores that were previously deposited by CARD-FISH staining. The protocol was tested on samples from an oligo-mesotrophic lake. Ultramicrobacteria affiliated with LD12 *Alphaproteobacteria* could be successfully sorted to high purity by flow cytometry. The ratios of median fluorescence signal to background ranged around 20, and hybridization rates determined by flow cytometry were comparable to those obtained by fluorescence microscopy. Potential downstream applications of our modified cell staining approach range from the analysis of microdiversity within 16S rRNA-defined populations to that of functional properties, such as the taxon-specific incorporation rates of organic substrates.

## Introduction

Flow cytometry has become an essential tool in aquatic microbiology (Wang et al., 2010). Individual microbial cells can be characterized, distinguished, and even physically sorted based on their fluorescence and light scattering properties. A wide range of fluorescent dyes are available for non-autofluorescent microbes. For example, DNA binding dyes allow a fast and accurate determination of total cell numbers as well as estimations of cell size and DNA content (Felip et al., 2007), and combinations of membrane permeable and impermeable dyes are used to determine physiological states of cells (Lopezamoros et al., 1995). When applied to complex bacterial communities those techniques are, however, limited to bulk analyses in which traits are assigned to operationally defined populations typically composed of taxonomically and functionally diverse species. Therefore, there is demand for taxon-specific labeling approaches that are compatible with flow cytometry. Various immunohistochemical tools are available for this purpose in clinical applications. Their application is however limited to well-characterized taxa with cultivated representatives (Alvarez-Barrientos et al., 2000).

Cultivation independent staining protocols such as fluorescence *in situ* hybridization (FISH) can overcome this limitation. Extensive databases of environmental 16S and 23S rDNAs such as SILVA or RDP (Pruesse et al., 2007; Cole et al., 2014) and various software tools facilitate the design of specific probes for most environmental bacteria (Ludwig et al., 2004; Yilmaz et al., 2011). FISH with directly labeled oligonucleotide probes however only performs reliably if the ribosome content of the target cells is high (Hoshino et al., 2008). This is not the case for most microbes in the pelagic zones of non-eutrophic waters, thus creating a need for signal amplification steps. Catalyzed reporter deposition (CARD) FISH is routinely applied for the microscopic quantification of such microbes (Pernthaler et al., 2002). This signal amplification procedure increases fluorescence intensities by 26–41 fold compared to standard FISH protocols (Hoshino et al., 2008; Stoecker et al., 2010). CARD-FISH and flow cytometry have been successfully combined for the sorting of planktonic marine bacteria with fairly large cell sizes and consequently high ribosome content (Sekar et al., 2004) and this combination has even been suggested for cell quantification in environmental samples (Manti et al., 2011). However, flow cytometry and CARD-FISH have so far never been applied to specifically target the smallest members of natural bacterioplankton communities (i.e., ultramicrobacteria) such as the LD12 *Alphaproteobacteria* or the freshwater acI *Actinobacteria* (Warnecke et al., 2005; Newton et al., 2011; Salcher et al., 2011), and it is unclear if CARD-FISH would provide sufficient signal intensities for this purpose.

Here, we present 2C-FISH, a modified FISH protocol based on sequential CARD that allows for flow cytometric sorting of small ultramicrobacteria that are not detectable by the previously described protocols. This was achieved by a second round of signal amplification with horseradish peroxidase labeled antibodies specific for the fluorophore previously deposited by CARD-FISH.

## Materials and methods

### Sampling

Lake Zurich is an oligo-mesotrophic prealpine lake with a maximal depth of 136 m (Posch et al., 2012). Samples were taken at the deepest point (N47°18′8.82″ E8°34′42.91″) on August 27 in 2010 and on May 18, June 22 and July 26 in 2011. After pre-filtration through 0.8 μm pore size membrane filters (Whatman) cells were fixed in formaldehyde (1.7% v/v) at 4°C for 15 h and collected on white membrane filters (GTTP02500, Millipore, diameter 25 mm, pore size, 0.22 μm). Twenty ml of sample was collected on each filter, which is approximately 5–10 times more than would be used for microscopic counting. The filtered volumes were optimized to collect a maximal number of cells while retaining a single cell layer. The filters were rinsed with particle-free deionized water, dried at room temperature and stored at −20°C.

### CARD-FISH (fluorescence *in situ* hybridization and catalyzed reporter deposition)

CARD-FISH and automated image analysis were conducted as described by Salcher and colleagues (Salcher et al., 2011). The following HRP-labeled oligonucleotide probes were used: BET42a, LD12-121 and NON338 (Manz et al., 1992; Wallner et al., 1993; Salcher et al., 2011). Additionally, the theoretical probe affinity of probe LD12-121 was analyzed *in silico* and optimized by the addition of six nucleotides (Yilmaz and Noguera, 2004; Yilmaz et al., 2011). The optimal stringency for the newly designed probe LD12-115 (5′-CTGAACCACAAGGCAGATTCCCACAT-3′) was determined on environmental samples, by increasing the concentrations of formamide in the hybridization buffer until the signal was lost. The most stringent conditions with acceptable fluorescence signal strength were at 60% of formamide.

### 2C-FISH (double CARD-FISH)

#### Initial CARD-FISH

The first parts of 2C-FISH were derived from the CARD-FISH protocol by Sekar et al. (2003) with the following modifications (see Table 1 for a step-by-step protocol): (i) Filters were not coated with agarose in order to facilitate subsequent cell removal. (ii) Lysozyme and achromopeptidase pre-digestion treatments were reduced to 30 and 20 min, respectively. (iii) The hybridization and incubations for tyramide signal amplification were conducted in 25 mm petri dishes containing 2 ml of the respective reagents. (iv) The first and second washing steps after hybridization were prolonged to 20 and 45 min. (v) The time of signal amplification using fluorescein labeled tyramides was increased to 30 min. (vi) After the washing step with PBS-T (Phosphate-Buffered Saline plus 0.01% Triton X-100) the filters were washed 2 more times in 1x PBS at 37°C for a total of 20 min and processed as described below while still wet.

**Table 1:** **Detailed 2C-FISH protocol**.

**PERMEABILIZATION**	
1	Incubate filters in 0.01 M HCl (RT, 10 min)
3	Incubate filters in freshly prepared lysozyme solution (37°C, 30 min)
4	Wash filters in PBS and MQ
5	Incubate filters in freshly prepared achromopeptidase solution (37°C, 20 min)
6	Wash filters in MQ and ethanol, air dry
**HYBRIDIZATION**	
7	Cover inside of small petri dish with parafilm (25 mm)
8	Place filter inside, add hybridization mix
9	Close and seal the lid with parafilm
10	Incubate (35°C, 2 h)
11	Prepare and preheat washing buffer
12	Wash filters in washing buffer (37°C, 20–30 min)
13	Incubate filters in PBS-T (45 min, 37°C)
**TYRAMIDE SIGNAL AMPLIFICATION**	
15	Prepare 0.15% H_2_O_2_
16	Mix 1 ml of amplification buffer + 10 μl 0.15% H_2_O_2_ + 2 μl fluorescein labeled tyramide
17	Dab filters onto blotting paper to remove excess liquid (don't let filters dry)
18	Incubate filters in tyramide solution in the dark (37°C, 30 min)
19	Wash filters in PBS-T (37°C, 10 min) and 2 times in PBS in the dark (37°C, 2x 10 min)
**ANTIBODY BINDING, SECONDARY CARD**	
20	Dilute antibodies 20–100 times with TNB buffer in a 500 μl reaction tube
21	Cut filters in 4 sections and place them in antibody solution
22	Incubate (4°C, overnight)
23	Wash 3x in PBS (37°C, 20 min total)
24	Prepare amplification mix (see above)
25	Incubate (37°C, 20 min)
26	Wash 3x in PBS (37°C, 20 min total)
**CELL REMOVAL**	
27	Cut each filter section in 4 pieces
28	Add 1.5 ml NaCl/Tween 80 mix to 2 ml reaction tube
29	Place filter sections inside, incubate in the dark (4°C, 20 min)
30	Attach the reaction tube to a vortex, vortex at full speed (RT, 15 min)
31	Transfer cell suspension to a fresh reaction tube and process within 24 h

*RT, room temperature*.

#### Antibody binding and secondary signal amplification

The filters were cut into 4 equal pieces and transferred to 0.5 ml reaction tubes (Eppendorf) containing 500 μl of 20–100x diluted Anti-Fluorescein-HRP conjugate (Roche, Switzerland) in Tris/NaCl/blocking buffer (TNB, 100/150 mM/1%). Antibody binding was conducted over night at 4°C, followed by 3 washing steps in 1x PBS for a total of 20 min at 37°C. A secondary tyramide amplification step again using fluorescein labeled tyramides was conducted in 2 ml reaction tubes (Eppendorf) at 37°C in a water bath for 20 min, followed by 3 washing steps in 1x PBS for a total of 20 min.

#### Cell removal

The cells were removed from the filters as previously described (Sekar et al., 2004). Briefly, each quarter of a filter was further cut into 4 sections, transferred to a 2 ml reaction tube (Eppendorf) containing 1.5 ml of NaCl/Tween 80 (150 mM/0.05%) and incubated at 4°C for 20 min. The reaction tubes were attached to a rotating shaker with adhesive tape (Vortex Genie 2 with 3 inch platform, Scientific Industries; Time Tape, Milian) and vortexed at full speed for 15 min. The cell suspension was transferred to fresh reaction tubes and processed on the same day. If larger debris particles were present, an additional filtration step was introduced (Swinnex filter holders, diameter 13 mm; Whatman polycarbonate filters, pore size 0.8 μm). A detailed step-by-step protocol of the whole procedure is presented in Table 1.

### Flow cytometry

Flow cytometry was conducted with an InFlux V-GS Flow Cytometer (Cytopeia Inc.) equipped with a blue laser (Sapphire HP, 200 mW, 488 nm wavelength, Coherent Inc.) and an UV laser (Lightwave Electronics, CY-PS, 60 mW, wavelength of 355 nm) controlled by a computer equipped with the software Spigot 5.2 (Pentium 4, 3 GHz, Windows 2000). Sheath pressure was set to 23 psi and sample pressure was adjusted to 1500–3000 events/sec. Side scatter, blue (460 ± 25 nm, DAPI) and green fluorescence (531 ± 20 nm, 2C-FISH/CARD-FISH) signals were analyzed in logarithmic amplification mode. Eight peak calibration particles (Sphero Rainbow Calibration Particles) were used to confirm the linearity of the instrument and to determine changes in channel numbers per decades of signal intensity at 531 ± 20 nm (Sharrow, 2001). Side scatter, 460 and 531 nm intensities were extracted to.csv files with FCSExtract 1.02 (http://research.stowers-institute.org/mcm/efg/ScientificSoftware/Utility/FCSExtract/index.htm). Standardized cytograms were produced by randomly selecting 100,000 events from each acquired sample. Thresholds for the calculation of signal to noise ratios were determined individually for each sample and set to the 99.5% quantiles of channel numbers in the corresponding negative controls (R version 3.1.2, pastecs package; R Development Core Team, 2011). Relative signal intensities (i.e., signal to beads ratios) were determined based on intensities of fluorescent beads recorded together with the samples (Flow Check^®^ YG, 1 μm diameter). For the determination of hybridization rates and for flow sorting, cells were counter-stained with DAPI (4′,6-Diamidino-2-phenylindole, 0.5 μg ml^−1^) and analyzed based on their blue and green fluorescent signals. Flow sorting was conducted in “two tube” mode (two-way sorting) with a preset of “Purity- Yield” (1 drop pre and post coincidence window, 1 drop sort mode). Target populations were selected in bivariate dot plots depicting green vs. blue fluorescence. For each sorting gate 50,000–250,000 cells were sorted and immediately processed for microscopic analysis.

### Microscopic evaluation of suspended CARD- and 2C-FISH stained cells

Subsamples of 70–100 μl of the sorted cell populations were spotted within a diameter of 5 mm on black membrane filters (Osmonics, pore size 0.22 μm, diameter, 25 mm). Filters were mounted on microscopic slides with mounting medium containing DAPI (Pernthaler et al., 2002) and analyzed at a Zeiss microscope (Zeiss Imager Z2) at 630X magnification (Zeiss Plan Apochromat, NA 1.4). Micrographs were produced by acquiring z-stacks of 7 images followed by image processing with the AxioVision module “Extended Focus” (Zeder and Pernthaler, 2009).

## Results and discussion

Freshwater ultramicrobacteria such as LD12 *Alphaproteobacteria* (also known as SAR11-IIIb, Salcher et al., 2011; Grote et al., 2012) or acI *Actinobacteria* (Warnecke et al., 2005) are characterized by a very small cell size, streamlined genomes, and high microdiversity (Zaremba-Niedzwiedzka et al., 2013; Ghylin et al., 2014). Although these microbes may numerically dominate freshwater microbial assemblages (Newton et al., 2011), attempts to obtain pure cultures have failed so far, thus making cultivation-independent approaches all the more indispensable. Moreover, taxonomy-based sorting (e.g., by flow cytometry) may be hampered by the very small cell size of ultramicrobacteria. We therefore developed a protocol that successfully combines population specific staining and flow sorting of ultramicrobacteria affiliated with LD12 *Alphaproteobacteria*.

### Development of 2C-FISH

**Figure 1:**
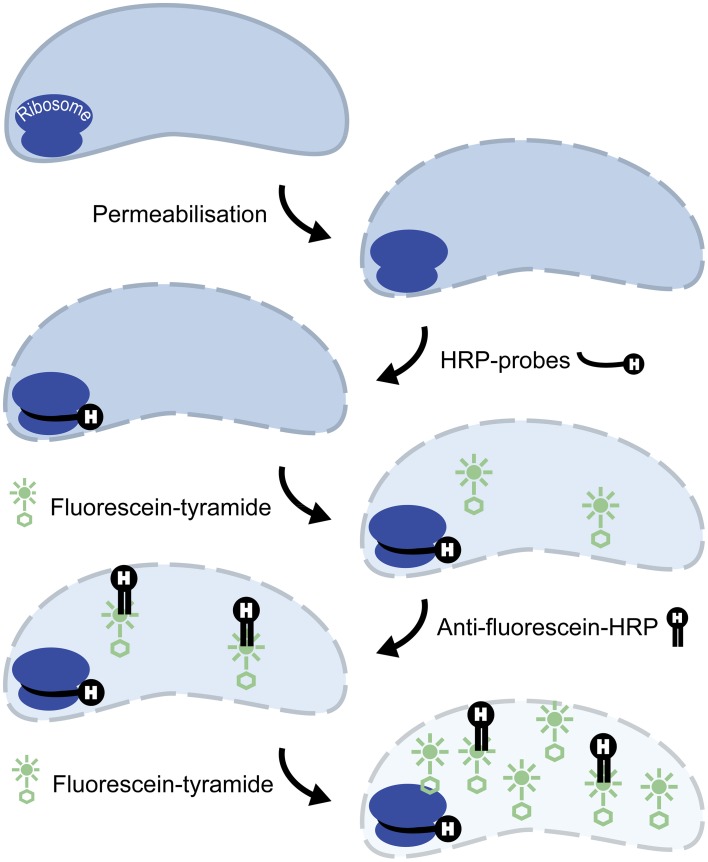
**Principle of 2C-FISH**. CARD-FISH step: Cell walls are permeabilized, horseradish peroxidase (HRP) conjugated oligonucleotide probes are hybridized to complementary sites of the ribosomes, and signal amplification is conducted with fluorescein labeled tyramides. Secondary signal amplification step: HRP conjugated anti-fluorescein Fab fragments are bound to the fluorophores deposited during the CARD-FISH step, and a second CARD signal amplification with fluorescein labeled tyramides is subsequently performed.

**Figure 2:**
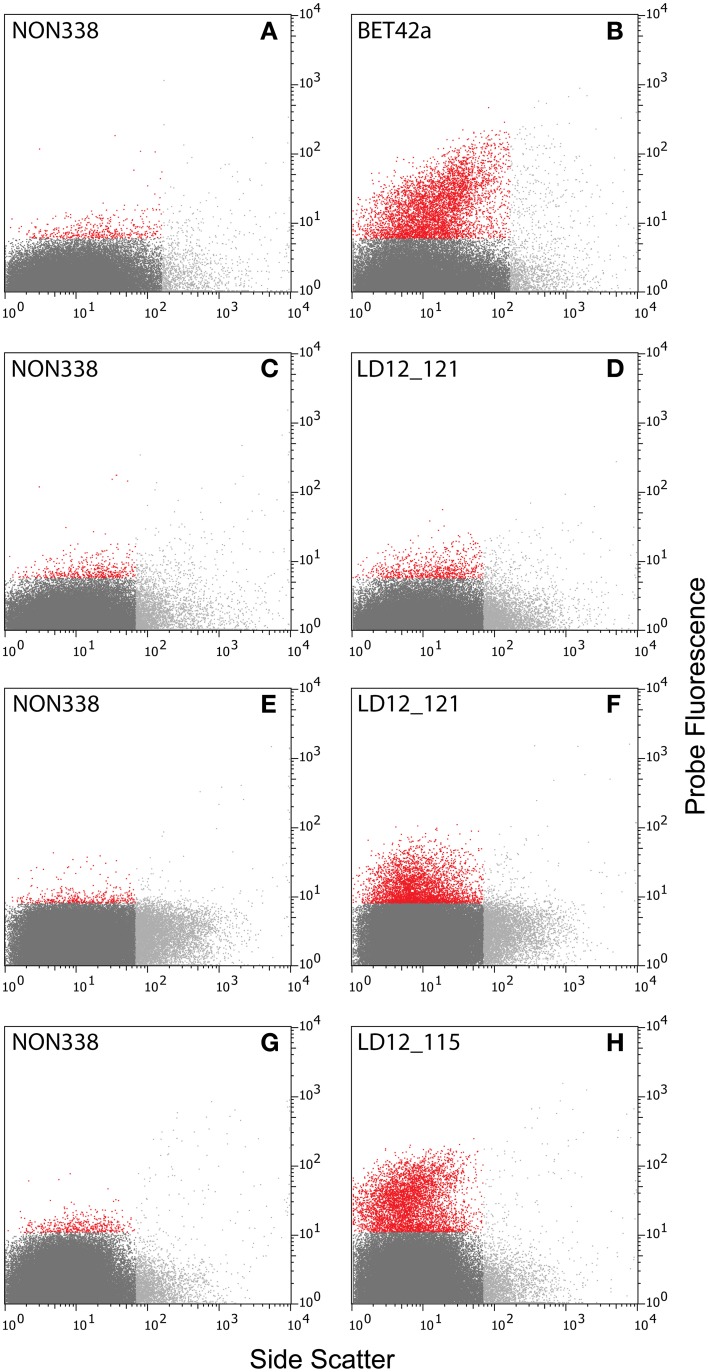
**Cytograms [probe fluorescence (520 ± 15 nm) vs. side scatter]. (A–D)** Natural bacterial assemblages after CARD-FISH with probes BET42a **(B)**, LD12-121**(D)**, and corresponding negative controls with probe NON338 **(A,C)**. **(E–H)** Natural bacterial assemblages after 2C-FISH with probes LD12-121 **(F)**, LD12-115 **(H)**, and corresponding negative controls **(E,G)**. Color codes: Light gray: Events with too large side scatter values that were excluded from analysis. Red and dark gray: Events with fluorescence intensities above and below the 99.5% quantile of the fluorescence intensities of the negative controls **(A,C,E,G)**.

**Figure 3:**
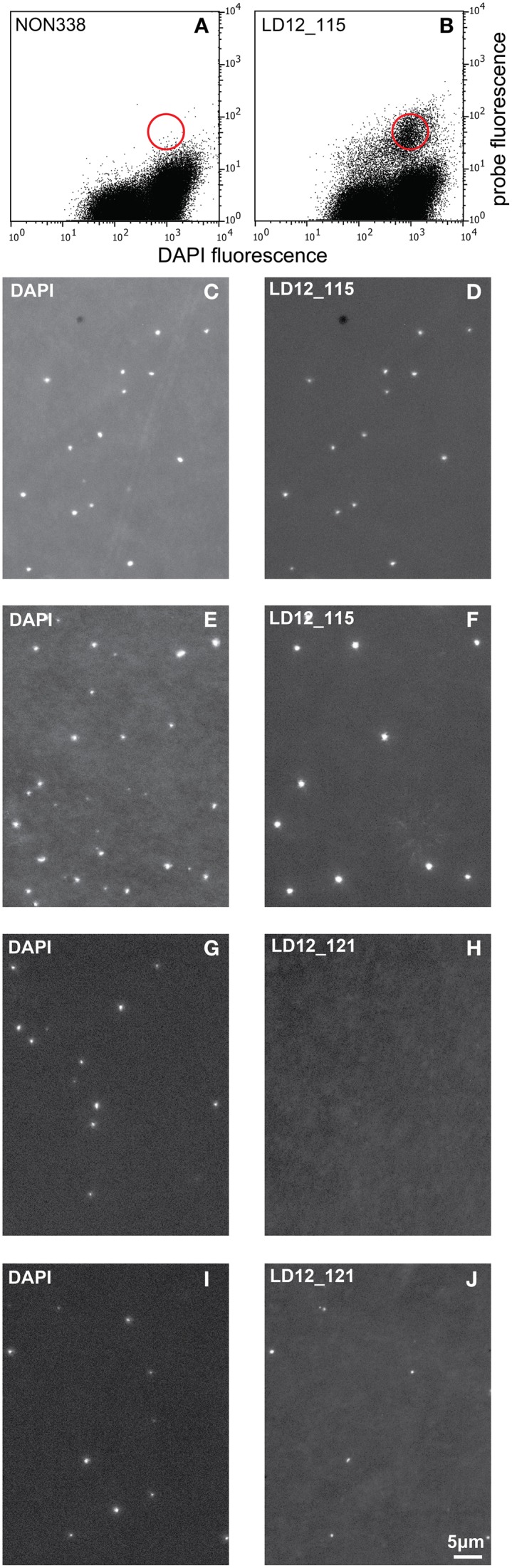
**Cytograms (probe fluorescence vs. DAPI fluorescence) and epifluorescence micrographs**. Cytograms of natural bacterial assemblages after 2C-FISH with the probes NON 338 **(A)** and LD12-115 **(B)**. The red circle symbolizes the approximate position of the sorting gate. Epifluorescence micrographs (left: DAPI fluorescence; right: Probe fluorescence). **(C,D)**: Cells sorted after 2C-FISH with the probe LD12-115. **(E,F)**: Natural bacterial assemblage after CARD-FISH with the probe LD12-115. **(G–J)**: Natural bacterial assemblage after CARD-FISH **(G,H)** and 2C-FISH **(I,J)** with the probe LD12-121 after resuspension of the cells.

Initial tests showed that conducting CARD-FISH for LD12 ultramicrobacteria directly in liquid phase was problematic (data not shown). The required centrifugation speeds (>14,000 × g) led to co-sedimentation of particles presumably from the blocking reagent and no detection of probe signals. We therefore conducted CARD-FISH on membrane filters and detached the cells at the end of the procedure (Figure 1). Microscopic evaluation confirmed successful labeling, however, the cell detachment procedure led to a substantial if not complete loss of signal (Figures 2D, 3H). Similar observations were made using a probe targeting substantially larger cells affiliated with *Betaproteobacteria* (BET42a). In this case, the remaining signal was however still sufficient for microscopic and flow cytometric detection (Figure 2B). One possible cause for this phenomenon may be cell permeabilization (Vives-Rego et al., 2000): Activated tyramides and soluble proteinaceous cellular components appear to leak out of the permeabilized cells and attach in their immediate proximity on the filter surface. While these compounds may still contribute to the overall fluorescence of a cell before the detachment procedure, this signal is lost once the cells are resuspended. In fact, a microscopic inspection of filters after cell detachment still yielded numerous cell-shaped spots with apparently CARD-FISH conferred fluorescence, albeit without corresponding DAPI signal (data not shown). Unfortunately, none of our attempts to overcome this issue by adjusting fixation and permeabilization conditions were successful, nor were increased hybridization and amplification times or increased concentrations of dextran sulfate, probe or labeled tyramide. We therefore aimed at increasing the number of fluorophore molecules bound per cell:

- Probe optimization: The rather low binding strength of the published probe LD12-121 (Salcher et al., 2011) was improved from a theoretical binding strength (ΔG°overall) of −14.0 to −18.2 kcal mol^−1^ by the addition of six nucleotides to the 5′-end (http://mathfish.cee.wisc.edu/probeaff.html). The modified probe (LD12-115, TGAACCACAAGGCAGATTCCCACAT) resulted in clearly improved signal intensities of individual cells (Figure 3F). At the same time, it did not change the total proportions of detected LD12 cells. The number of sequences targeted by the original and modified probe in the SILVA database (SSU r121 PARC, Pruesse et al., 2007), was 843 and 833, respectively, i.e., both probes covered 91–91.9% of all sequences affiliated with LD12 *Alphaproteobacteria*. The number of outgroup hits with 0 mismatches was 26 for both probes, and the modified version had substantially less outgroup hits with one mismatch (1 vs. 46). Probe LD12-115 therefore represents a superior option for quantifying LD12 *Alphaproteobacteria* in environmental samples.
- Second CARD layer: Cells labeled with the new probe were still not sufficiently detectable by flow cytometry after removal from the filters (data not shown). Therefore a second signal amplification step similar to the one described by Kubota et al. (2006) was introduced (Figure 1). However, we replaced the anti-DNP staining system suggested by those authors with an anti-fluorescein detection system that made use of fluorescein-labeled tyramide in both amplification steps. Besides reducing the number of required reagents, our approach allows for the contribution of both amplification steps to the final fluorescent signal of cells. This strategy substantially increased the fluorescence signals of the suspended cells, to levels that were sufficient, both, for microscopy and flow cytometry (Figures 2F, 3J). If combined with the optimized probe LD12-115, the signal intensities and signal to noise ratios were comparable to those of CARD-FISH stained *Betaproteobacteria* (Figures 2H, 3B,D; Table 2). *Betaproteobacteria* in Lake Zurich are typically large and fast growing cells (e.g., *Limnohabitans* sp., Eckert et al., 2012) that generally show bright CARD-FISH signals (Figure 2B; Table 2). In this context we also analyzed a culture of the freshwater strain *Limnohabitans* sp. RIM47 (Kasalický et al., 2013) after CARD-FISH and 2C-FISH staining by flow cytometry (see Supplementary Information). However, no significant improvement of the signal to noise ratio of 2C-FISH as compared to CARD-FISH was observed (data not shown). This suggests that the 2C-FISH approach is useful for very small cells, but does not provide additional advantage for bacteria that can be sufficiently stained for flow cytometric analysis by CARD-FISH only. By contrast, a population of acI *Actinobacteria* from Lake Zurich could only be visualized by flow cytometry after 2C-FISH staining (Supplementary Figure S1).

**Table 2:** **Overview of analyzed samples and quantitative information on hybridization efficiency**.

**Sample**	***n***	**Probe**	**S/N FC 2C-FISH**	**Rel. int. FC (x 10^−3^) 2C-FISH**	**%Hyb FC 2C-FISH**	**%Hyb Mic CARD FISH**
27.08.2010	2	Bet42a	18.34 ± 0.08*	1.79 ± 0.01*	n.d.	5.7
27.08.2010	2	LD12-121	n.d	n.d	n.d.	10.4
27.08.2010	3	NON-338	n.d	n.d	n.d	n.d
18.05.2011	5	LD12-121	6.97 ± 1.23	1.57 ± 0.08	n.d.	22.4
18.05.2011	3	NON-338	n.d	n.d	n.d	n.d
22.06.2011	2	LD12-115	16.66 ± 4.35	2.15 ± 0.56	13.45 ± 0.91	15.7
22.06.2011	2	NON-338	n.d	n.d	0.03 ± 0.00	n.d
26.07.2011	3	LD12-115	26.47 ± 12.69	2.90 ± 1.14	5.97 ± 1.32	10.9
26.07.2011	3	NON-338	n.d	n.d	0.19 ± 0.12	n.d

* *CARD-FISH was used for all analyses with probe Bet42a; S/N FC, Signal to noise ratios determined by flow cytometry; Rel. int. FC, Intensities relative to those of fluorescent beads; %Hyb FC, Hybridisation rates determined by flow cytometry; %Hyb Mic, Hybridisation rates determined by microscopy; n.d, not determined*.

### Potential applications and limitations of 2C-FISH

While we did not aim to develop a protocol for a flow cytometric quantification of ultramicrobacterial populations in water samples, we nevertheless compared the relative abundances of LD12 in Lake Zurich water samples as either determined by our new approach in combination with flow cytometry or by CARD-FISH in combination with microscopy. Generally, the differences between 2C-FISH stained replicates were low (coefficient of variation ≤0.22) and the agreement between flow cytometric (2C-FISH stained) and microscopic (CARD-FISH stained) determinations of cell proportions were good to excellent (Table 2). However, since the preparation of 2C-FISH samples is also considerably more time-consuming than CARD-FISH, we see the main potential of this approach in its combination with flow sorting based applications.

CARD-FISH stained and sorted cells have been successfully used as templates for PCR amplicon sequencing (Sekar et al., 2004). The same holds true for cells stained by 2C-FISH, since no additional chemicals apart from FAB fragments are involved in this procedure. One potential application could, therefore, be a population wide assessment of the diversity of selected functional genes (e.g., of rhodopsin genes, Martinez-Garcia et al., 2012) or of phylogenetic markers with higher resolution than 16S rRNA genes (Whitaker et al., 2003). Unlike community-level amplicon sequencing or sequence similarity based recruitment from metagenomes, such an approach might be more robust against erroneous assignments due to recent events of horizontal gene transfer.

Other molecular approaches such as producing population metagenomes from sorted cells can also be envisaged, but might require prior adaptations of the here presented protocol. Specifically, the limited yield of a targeted cell enrichment by flow sorting (depending on in situ densities, between 10^5^–10^6^ cells can be obtained) would make whole genome amplification (WGA) inevitable (Blainey, 2013). However, current WGA methods are not compatible with formaldehyde fixation (Clingenpeel et al., 2014). Therefore, alternative fixation protocols (e.g., using ethanol) might be necessary in order to amplify genomic material from flow sorted 2-C stained ultramicrobacteria. As a proof of principle, we sorted formaldehyde- and ethanol-fixed cells of the freshwater strain *Limnohabitans* sp. RIM47 after 2C-FISH staining. We first subjected the sorted cells to WGA. Subsequently we could amplify 16S rRNA genes from WGA products (Supplementary Figure S2), as confirmed by sequence analysis (see Supplementary Material).

Another potential application might be to quantify the uptake of radiolabeled organic compounds in flow sorted 2C-FISH stained ultramicrobacteria, as has been done for methionine in autofluorescent marine picocyanobacteria (Zubkov et al., 2003). A precise determination of the taxon-specific incorporation rates of organic substrates could provide valuable information about the ecophysiology of bacterial populations (e.g., their uptake kinetics) and about their contribution to the total microbial utilization of these compounds. This would add quantitative information to the different substrate utilization patterns of freshwater microbes as revealed by CARD-FISH and microautoradiography (Salcher et al., 2013), and it might eventually allow for the testing of the increasingly detailed hypotheses generated from genomic data, e.g., carboxylic acid vs. carbohydrate specialization of LD12 *Alphaproteobacteria* and acI *Actinobacteria*, respectively (Zaremba-Niedzwiedzka et al., 2013; Ghylin et al., 2014). In summary, our approach will add to the spectrum of available single-cell tools for the study of aquatic ultramicrobacteria such as LD12 *Alphaproteobacteria*, and it might help to gain new insight into their diversity and ecophysiology.

### Conflict of interest statement

The authors declare that the research was conducted in the absence of any commercial or financial relationships that could be construed as a potential conflict of interest.
