# Supplementary material for: Fluorescence in situ hybridization and sequential catalyzed reporter deposition (2C-FISH) for the flow cytometric sorting of freshwater ultramicrobacteria
Source: Front Microbiol. 2015 Mar 31;6:247. doi: 10.3389/fmicb.2015.00247 (PMC4379941; doi:10.3389/fmicb.2015.00247)
Supplement: Supplementary file 1 [file Image1.PDF]

**Supplementary Figures:**

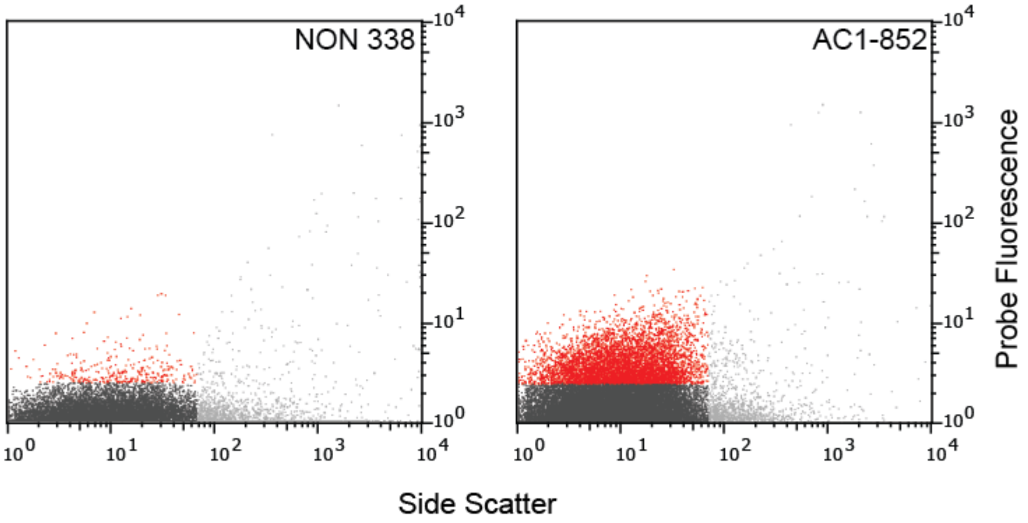


**Figure S1: Cytograms of natural bacterial communities stained with 2C-FISH using the probes NON338 (Control) and AC1-853 (acI *Actinobacteria*).** Plots depict probe fluorescence (520 ±15nm) vs. side scatter. The samples were collected on June 22, 2011 from the epilimnion of Lake Zurich. Colour codes: Light grey: Events with too large side scatter values were excluded from the analysis. Red and dark grey: Events with fluorescence intensities above and below the 99.5% quantile of the fluorescence intensities of the negative controls.

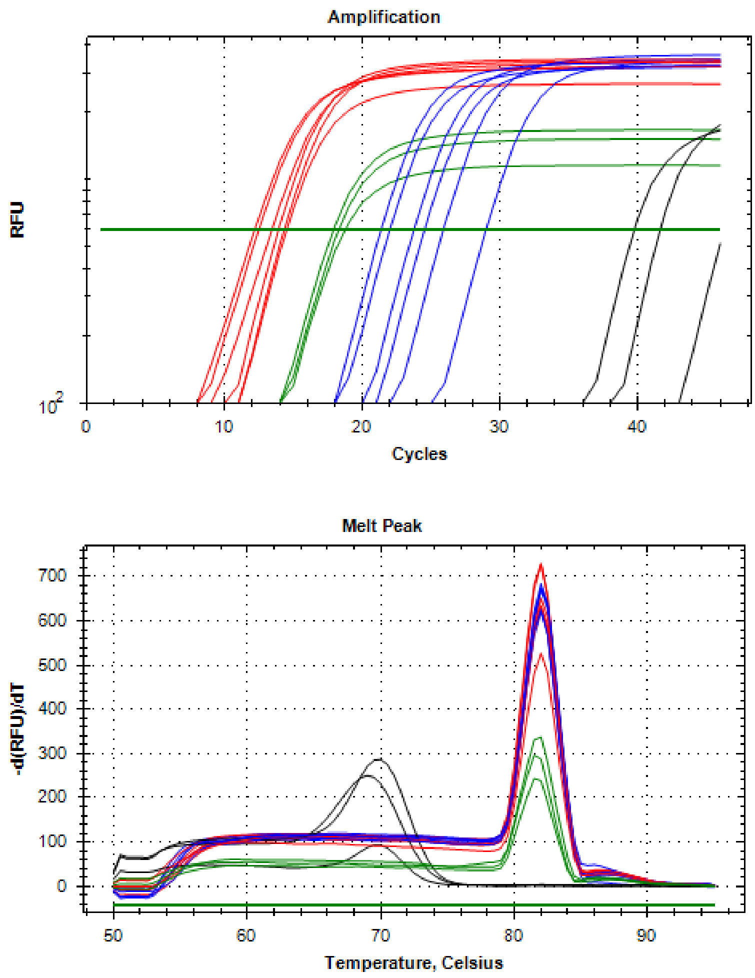


**Figure S2: PCR amplification curves (top) and corresponding melting curves (bottom) for *Limnohabitans* sp. RIM47:** Green: extracted DNA (control). Red and Blue: Ethanol and Formaldehyde fixed 2C-FISH stained *Limnohabitans* sp. RIM47 cells collected by flow cytometric sorting, respectively. Black: Negative controls.
