# Supplementary material for: Fluorescence in situ hybridization and sequential catalyzed reporter deposition (2C-FISH) for the flow cytometric sorting of freshwater ultramicrobacteria
Source: Front Microbiol. 2015 Mar 31;6:247. doi: 10.3389/fmicb.2015.00247 (PMC4379941; doi:10.3389/fmicb.2015.00247)
Supplement: Supplementary file 2 [file Presentation1.PDF]

**Supplementary Information:**

## Experiments with pure cultures of *Limnohabitans* sp. Rim47

*Methods:*

*Limnohabitans* sp. Rim47 (Kasalický et al., 2013) was cultivated on R2 medium at 18°C for 60 h. Aliquots (1 ml) were taken and diluted 10 times in ultrapure water. Half of the aliquots were amended with formaldehyde (1.7% v/v) and incubated for 15 hours at 4°C. After fixation 200 µl of the diluted culture were collected on membrane filters (Millipore GTTP2500, diameter 25 mm, pore size, 0.22 µm), air dried and stored at -20°C. The remaining aliquots were filtered as described above, incubated in ice cold ethanol and 1 x PBS (50:50, v:v) for 50 min, washed briefly in 1x PBS and 100% ethanol, air dried and stored at -20°C.

2C-FISH was conducted with probes BET42a and NON338 (Manz et al., 1992; Wallner et al., 1993). Half a million cells per treatment were sorted and centrifuged at 10,000 × g for 20 minutes. The supernatant was removed and the cells were stored at -20°C until further processing.

Multiple displacement amplification (MDA) was conducted with a *REPLI-g* SC kit (Qiagen) with the modification that the reaction volume was reduced to 12.5 µl and SYBR Green I (life technology) was added in a final concentration of 0.1 x. Six MDA and 5 PCR reactions were produced out of each sample containing approximately 500,000 sorted cells in 8 µl of sheet fluid. MDA products were diluted 100 times for PCR amplification and DNA extracts derived from untreated *Limnohabitans* sp. RIM47 cells were diluted to 2 ng/µl and served as control.

PCR with real-time monitoring was conducted with FastStart Universal SYBR Green Master (Roche) to allow a comparison between the effects of the two fixation procedures. The following 3 step amplification protocol was used: Activation of the polymerase was conducted at 95 °C for 10 min, followed by 45 cycles including a denaturation step at 95 °C for 15 s, an annealing step at 50 °C for 30s and an extension step at 60 °C for 45 s. Melting analysis was conducted between 50 – 95°C with 0.5°C increments for 5 s for each step.

PCR amplification of 16S rDNA was conducted with a GoTaq Green Master Mix (promega) with the primers GM1f and GM3r (Muyzer et al., 1993; Muyzer et al., 1995) according to the manufacturers' protocol. The PCR products were purified with a High Pure PCR Product Purification Kit (Roche) followed by sequencing with the ABI BigDye chemistry on an ABI 3730 Genetic Analyzer (Applied Biosystems) with the primer GM1f.

*Results:*

16S rRNA genes could be amplified from all MDA products irrespective of fixation, but PCR was substantially more effective with templates derived from ethanol fixed samples (Supplementary Figure S2). All 6 ethanol and 5 out of 6 formaldehyde fixed variants produced clean 16S rRNA gene sequences of *Limnohabitans* sp. RIM47. Sequencing of PCR products from unamplified samples was successful in 4 out of 5 cases irrespective of the fixation procedure.

*References:*

Kasalický, V., Jezbera, J., Hahn, M.W., and Šimek, K. (2013). The diversity of the *Limnohabitans* genus, an important group of freshwater bacterioplankton, by characterization of 35 isolated strains. PLoS ONE 8, e58209.

Manz, W., Amann, R., Ludwig, W., Wagner, M., and Schleifer, K.H. (1992). Phylogenetic oligodeoxynucleotide probes for the major subclasses of proteobacteria: Problems and solutions. Syst. Appl. Microbiol. 15, 593-600.

Muyzer, G., Dewaal, E.C., and Uitterlinden, A.G. (1993). Profiling of complex microbial populations by denaturing gradient gel electrophoresis analysis of polymerase chain reaction-amplified genes coding for 16S rRNA. Appl. Environ. Microbiol. 59, 695-700.

Muyzer, G., Teske, A., Wirsen, C.O., and Jannasch, H.W. (1995). Phylogenetic relationships of Thiomicrospira species and their identification in deep-sea hydrothermal vent samples by denaturing gradient gel electrophoresis of 16S rDNA fragments. Arch. Microbiol. 164, 165-172.

Wallner, G., Amann, R., and Beisker, W. (1993). Optimizing fluorescent in situ hybridization with rRNA-targeted oligonucleotide probes for flow cytometric identification of microorganisms. Cytometry 14, 136-143. doi: 10.1002/cyto.990140205
