# Supplementary material for: Fluorescence in situ hybridization and sequential catalyzed reporter deposition (2C-FISH) for the flow cytometric sorting of freshwater ultramicrobacteria
Source: Front Microbiol. 2015 Mar 31;6:247. doi: 10.3389/fmicb.2015.00247 (PMC4379941; doi:10.3389/fmicb.2015.00247)
Supplement: Supplementary file 3 [file Table1.PDF]

Supplementary Tables

Table S1: R script used for plotting of flow cytometric data

```
library(pastecs)

# required input: paths to sample and negative control (lines 11, 22)
# set manualy:    cut-off beads (lines 18, 29)
#                 threshold side scatter (lines 45, 53)

rm(list=ls())

# prepare sample

probe <- read.csv("E:/sample.csv", header=FALSE)[-c(1),]

probe$V1<-as.numeric(as.character(probe$V1))
probe$V2<-as.numeric(as.character(probe$V2))
probe$V3<-as.numeric(as.character(probe$V3))
row.names(probe)<-NULL

probeNB<- subset(probe,V2 <= 52835)

# prepare negative control

non <- read.csv("E:/negcontrol.csv", header=FALSE)[-c(1),]

non$V1<-as.numeric(as.character(non$V1))
non$V2<-as.numeric(as.character(non$V2))
non$V3<-as.numeric(as.character(non$V3))
row.names(non)<-NULL

nonNB<- subset(non,(V2 <= 52835))
nonQ <- subset(nonNB,V2 <= quantile(V2,0.995))

# subsample randomly

probe100k <- probeNB[sample(nrow(probeNB),size=99981,replace=FALSE),]
row.names(probe100k)<-NULL

non100k <- nonNB[sample(nrow(nonNB),size=99981,replace=FALSE),]
row.names(non100k)<-NULL

# plot sample:

par(pty="s")
plot(probe100k$V1, probe100k$V2, pch=".",
     main="",xlim=c(0.1,65536),ylim=c(0,65536),
     col=ifelse(probe100k$V1 >= 30000 , "grey68",
                ifelse(probe100k$V2 >= max(nonQ$V2), "red", "grey30")))

# plot non:

par(pty="s")
plot(non100k$V1, non100k$V2, pch=".",
     main="",xlim=c(0.1,65536),ylim=c(0,65536),
     col=ifelse(non100k$V1 >= 30000 , "grey68",
                ifelse(non100k$V2 >= max(nonQ$V2), "red", "grey30")))
```

Table S2: R script used for determining median signal intensities

```
library(pastecs)

# required input: paths to sample.csv and negative control.csv
(lines 11, 22)
# set manually:    cutoff side scatter and beads (lines 18, 29)


rm(list=ls())

# prepare sample

probe <- read.csv("E:/sample.csv", header=FALSE)[-c(1),]

probe$V1<-as.numeric(as.character(probe$V1))
probe$V2<-as.numeric(as.character(probe$V2))
probe$V3<-as.numeric(as.character(probe$V3))
row.names(probe)<-NULL

probeS <- subset(probe,(V1 <= 30000 & V2 <= 52835))

# prepare negative control

non <- read.csv("negcontrol.csv", header=FALSE)[-c(1),]

non$V1<-as.numeric(as.character(non$V1))
non$V2<-as.numeric(as.character(non$V2))
non$V3<-as.numeric(as.character(non$V3))
row.names(non)<-NULL

nonS <- subset(non,(V1 <= 30000 & V2 <= 52835))
nonQ <- subset(nonS,V2 <= quantile(V2,0.995))


# subset sample

target <- subset(probeS,(V2 >=  max(nonQ$V2)))
nontarget <- subset(probeS,(V2 <  max(nonQ$V2)))

#stats

stat.desc(target$V2)
stat.desc(nontarget$V2)
stat.desc(nonS$V2)
```
